# Monthly Digest for February

**Published:** 1889-03

**Authors:** 


### MONTHLY DIGEST.

FOR FEBRUARY.

February *Cosmos*, *Ohio Journal*, *Archives*, *Items of Interest*; *also* January *Review*, *Western Journal*, *Southern Dental Journal*, *and Headlight* received too late for notice in February issue.

### Operative Dentistry.

### PREPARATION OF THE MOUTH.

*The Dental Headlight* (January) publishes a paper read by Prof. W. H. Morgan before the Tennessee State Dental Society, on the importance of the treatment of adjacent parts preparatory to filling teeth, a large proportion of failures being due to the unhealthy condition of the soft tissues and consequent vitiated oral fluids. Soft, flabby gums pour forth viscid secretions, often acid; they also retain particles of finely comminuted food which ferment and produce other acids. This condition of the gums may be due to calculi, with which there may also be a discharge of pus or ichor.

Large V-shaped separations may have come together again, folding up and pinching the gum and making it unhealthy. These vitiated secretions, from whatever cause, have chemical qualities which break down tooth structure causing decay especially at the margin of the gum, and on approximate surfaces.

All these modifying conditions should be controlled to the benefit and interest of the patient in enhancing the value of fillings. There is an implied obligation to our patients to give them the benefit of all the skill we possess, and this obligation reaches beyond the operation especially in hand. Dr. Morgan is so emphatic on this point that, in examination papers he marks a student ten per cent. less on *graduation fillings* in a mouth where he finds salivary calculus!

### RESTORATION OF TOOTH CROWNS.

Dr. J. C. Walton, Howell, Michigan, in the January *Review*, describes a case of very extensive restoration of abraded tooth-crowns, including the superior central incisors, right lateral and cuspid, and the lower molars and bicuspids. The teeth were all worn very considerably, especially the lower molars and the upper incisors. The pulp chambers of several were filled with plugs of reparative dentine which looked like cylinders of amber. Approximal cavities in the molars afforded anchorage, but grooves had to be cut in the bicuspids, cuspid, and incisors. The fillings were started with mats of non-cohesive gold and built out with cohesive No. 60, to which was welded gold-and-platinum foil No. 60 with which the buildings were all completed, each tooth being completed at a sitting. Six years later the work was giving good satisfaction.

### A PORCELAIN TIP.

Mr. Skeet, New Zealand, in the *British Journal of Dental Science*, republished in the Jan. *Southern Dental Journal*, details the method employed in affixing a porcelain tip to a superior central incisor with living pulp, three-sixteenth of an inch having been broken off by an accident. The tip and the root were ground transversely to form a perfect joint; on the lingual surface of the tooth a groove was formed to receive a tongue of gold soldered to the backing of the tip, with undercuts for anchorage of gold filling, a small cavity being also cut near the gum connected with the larger cavity forming a dovetail for the reception of the end of the gold tongue. A little thin oxychloride was laid along the cavities and the joint. The tip and the tongue was then set in place and held firmly till the cement had set. Gold was then built up over tongue and backing, restoring the original shape of the tooth. This has now done good service for three years without the slightest discomfort.

### ROOT CANAL FILLING.

The editor of the *Western Journal* decrys earnestly the practice of filling root canals with cotton, which was openly advocated by several speakers at the recent meeting of the Odontological Society of Philadelphia.

Oxychloride cement is advocated as the best material for this purpose; *first*, because of the perfect union between the filling material and dentine; *second*, because it cauterizes and makes fixed material of any organic material left in the canal and dentinal tubes; and *third*, because it makes a better foundation for a gold filling. The objection that it may be forced through the foramen is overcome by using a small point of gutta-percha at the apex. Chloro-percha is objectionable because of shrinkage on evaporation.

Dr. H. C. Herring, Concord, N. C., in Jan. *Archives*, gives the very unfortunate termination of a case of immediate root filling in two superior centrals resulting in a typical case of alveolar abscess, the tissues all broken down, pus exuding from different points both in the palatal arch and the labial region, both teeth finally dropping out with pieces of bone coming away daily. From this and other cases he deduces the warning to young men not to "monkey" with new ideas gathered at meetings when too often there seems to be a craze about some new method judged by the apparent success of a few isolated cases to which the only real test, that of time, has not been applied.

PULP CAPPING.

Dr. E. R. Mullett, Clinton, Iowa, as reported in the *Items of Interest*, advocates capping exposed pulps, finding in his own practice that he has to extract a greater number of teeth whose roots have been treated and filled, than of teeth with properly capped nerves.

He caps with oxyphosphate, using if necessary a piece of quill cut to size and shape indicated. If the pulp is congested he bleeds it slightly and covers it for two or three days with cotton wet with listerine or oil of cloves, with a trifle of morphine added, and then caps as before.

ALVEOLAR ABSCESS.

Dr. I. P. Wilson, in the *Review*, describes a case of alveolar abscess discharging into the pharynx. After excessive swelling of the neck and fauces, clinched jaws, obstructed deglutition, and imminent danger of suffocation, a large quantity of pus was suddenly discharged nearly causing death from strangulation. The discharge continuing for two or three months various causes being assigned for the trouble, some one finally suggested that *possibly* the teeth might have something to do with it, and the case came into Dr. Wilson's hands. He found that a third inferior molar had been filled over a capped nerve, and discovered the ontlet of a drainage tube or fistulous tract leading from the roots of the molar to the pharyngeal cavity, the pus—instead of having been driven between the wall of the alveolus and the root to the neck of the tooth, or gravitating down the fibres of the internal pterygoid muscle, finding an outlet on the face, had evidently been conducted by the fibrous layers of the steylo-hyoid to the cavity of the pharynx.

The extraction of the molar, which had no antagonist, resulted in a speedy cure of the local trouble and improvement of the general health.

The Jan. *Western Journal* quotes a *Review* editorial on the excision of the ends of roots in the treatment of chronic alveolar abscess. When, after apparently thorough treatment, a root is filled and still the fistulous opening does not close and after the lapse of two weeks or a month the filling is removed and treatment and filling is again followed by failure to heal, examination of the end of the root through the fistula will probably reveal the fact that the apex is roughened or partially if not completely denuded of peridental membrane. Then excision of the apex is clearly indicated or serumal deposits may encircle the apex and the abscess will not heal till these are removed, with or without the apex. Or there may be some absorption of the root, or the pulp may have died before completion of the root. These are some of the causes that indicate excision of the root. The locality of the root will indicate the method of opening the gum and alveolar process. All debris should be washed away with bichloride of mercury or five per cent. resorcian. The wound should be dressed with iodoform, iodol or hydronapthol, or if very extensive, packed with hydronapthol-cotton for a day or two.

The compiler of *Memoranda* in the *Review* says that he has twice recently seen a pulpless lateral and central incisor with alveolar abscesses discharging through a common fistulous opening. He had also recently drilled into a superior first bicuspid finding the bulbous portion of the pulp and in the canal of the labial root dead with abscess and fistulous opening, the palatal root pulp being alive. He found a gold filling had impinged on the labial coruna of the pulp for several years.

ANTRAL ABSCESS.

The *Cosmos*, February, publishes an exhaustive paper from Dr. Frank Abbott, on Disease of the Antrum, Due to Dental Complications, as also the discussion of the paper in the New York Odontological Society, where it was read. The paper embraces the history, location, physiology, and function of the antrum, with diagnosis, differential diagnosis, prognosis, and treatment of the disease which he considers as an alveolar abscess, the pus from which, finds exit into and through the antrum. The mucous membrane lining the antral cavity becomes inflamed and hypertrophied from the presence of the pus ulceration taking place, if of long standing. The means of diagnosis and of differential diagnosis are clearly and fully indicated by Dr. Abbott. In his treatment, after obtaining a suitable opening for exploration, application of remedies, and the involuntary evacuation of fluid contents, permanganate of potash is used to control unpleasant odors, and the cavity is washed with carbolic acid 1 to 64, or listerine and water equal parts; a saturated solution of boracic acid and warm water is also very effective. Bichloride of mercury he finds too irritating except 1 to 5,000. After washing, the cavity is also thoroughly sprayed in all directions with the remedy selected. The opening is kept free by means of a tent dipped in carbolized oil of sweet almonds, 1 to 15, the tent being tied to an adjoining tooth.

Peroxide of hydrogen may be used to advantage to test the presence of pus. If a specific poison has to be combated, iodide of potassium or sulphide of calcium is indicated; also ammonia-citrate of iron for anemic patients.

Dr. R. M. Streeter is afraid of over-medication. He uses hydronapthol for cleaning, iodine and alcohol as local stimulant, and a weak solution of chloride of zinc in case of much ulceration.

Dr. Dwinelle described some very extreme cases illustrating the "ignorance of the learned" in the medical profession, which yielded readily to treatment from a dental standpoint. In one case the opening was so very large that with a curved lancet he cut the tissue around the cavity bringing it down in the shape of a collar or ruffle; the raw surfaces which he brought in contact were secured with a Marion Sims silver suture.

Dr. Lord uses a weak solution of phenol sodique.

In the Iowa State Society, reported in Feb. *Items of Interest*, Dr. E. D. Brower read a paper covering the same ground but with less fullness of detail. His treatment consists simply in opening up thoroughly and washing out with warm water followed by listerine or corbolized water, forty drops to the pint.

NECROSIS.

Dr. L. L. Davis reports in the Jan. *Review* a case of syphilitic necrosis presenting some interesting features. The gums were swollen and spongy an exudate of purulent matter surrounding each tooth, all of which were more or less loose, but free from caries. The upper right first and second molars had been extracted, and from this point, from the middle of an ulcer, a small hard mass projected surrounded by abundant purulent secretions. The same condition, but in less degree, existed in the lower jaw where the second and third molars had been extracted. He was under systemic treatment which was continued. In the lower jaw the remaining molar was removed and all necrosed bone, which included both outer and inner alveolar plates from a point a little posterior to the second bicuspid to the angle of the ramus, measuring one and-a-half inches. The necrosed bone in the upper maxilla was the size of a large hazel nut and included a portion of the floor of the antrum. Hypodermic injections of cocaine were used and the patient suffered very little pain. Within two weeks the lower jaw was in a healthy condition. The opening into the antrum is now slowly closing.

PYORRHŒA ALVEOLARIS.

The *Ohio Journal* continues the republication of Frank Lankester's paper on some affections of the gums. Of pyorrhœa alveolaris he says that it is an obscure though important and not very uncommon affection, of which the causes and pathology are very obscure, with very good reasons for considering it in part a constitutional disease. The treatment includes thorough removal of tartar, the use of caustics—as powdered sulphate of copper, iodoform, aromatic sulphuric acid, chloride and iodide of zinc, etc.

Dr. C. A. Timme, in the First District Society, New York, as reported in the *Cosmos* demonstrated the application of the thermo-cautery in the treatment of pyorrhœa alveolaris. Hollow-tube platinum points are attached to a special hand piece which is connected with an atomizer containing naphtha. The point is heated over an alcohol flame and kept at a white heat from the effect of the naphtha spray.

Dr. M. L. Rhein read before the Union Convention of the Fifth, Sixth, Seventh, and Eighth District Society of New York, (*Cosmos*) a paper on this subject. He says that, as a rule, when results are not satisfactory it will be found that patients have not kept the teeth properly cleaned. In old chronic cases where the teeth are almost ready to tumble out of their sockets, reproduction of tissue can not take place unless they are held immovably in place. This can be accomplished by cutting a groove in the teeth and laying a bar of gold in it, or building a continuous gold filling all around. Dr. Rhein says that properly constructed

RRIDGEWORK

Can be anchored to teeth in the last stage of pyorrhœa alveolaris, and the patient will have the benefit of the bridge while the diseased teeth being firmly held in position can be easily brought into a healthy condition.

"Free Lance," the New Jersey correspondent of the *Review* speaks in the highest terms of Dr. Parr's removable bridgework. The crowns or caps for anchorage are made as usual, gold bands being attached which form longitudinal slots or grooves on each cap, into which a heavy spring is inserted making the work firm and rigid while readily removable by the patient. The value of this feature, from a hygienic point of view, will be readily recognized. Dr. Parr gives this freely to the profession asking only a just recognition of his services.

Dr. J. G. Harper (*Archives*) dubs himself "a doubting Thomas," on the subject of bridgework pronouncing it a physical mpossibility for one member to do the work of half a dozen.

CROWNS.

Dr. J. Morgan Howe gave the New York Odontological Society (*Cosmos*) the following method of setting porcelain crowns on very frail, thin roots which are liable to split. A narrow band is fitted around the neck of the root to which is soldered a cap through which passes a pivot. Vent holes are drilled through the cap, which is set in place with oxyphosphate, any excess being forced through the holes instead of around the band under the gums. When the cement has set it is reamed out through the holes which are then filled with gold. If the crown should give way it is easily replaced without disturbing the foundation.

